# Human matters in asthma: Considering the microbiome in pulmonary health

**DOI:** 10.3389/fphar.2022.1020133

**Published:** 2022-12-02

**Authors:** Sandesh J. Marathe, Mark A. Snider, Armando S. Flores-Torres, Patricia J. Dubin, Amali E. Samarasinghe

**Affiliations:** ^1^ Department of Pediatrics, College of Medicine, University of Tennessee Health Science Center, Memphis, TN, United States; ^2^ Division of Pulmonology, Allergy-Immunology, and Sleep, Memphis, TN, United States; ^3^ Children’s Foundation Research Institute, Le Bonheur Children’s Hospital, Memphis, TN, United States; ^4^ Division of Emergency Medicine, College of Medicine, University of Tennessee Health Science Center, Memphis, TN, United States

**Keywords:** diet, smoking, therapeutics, pediatric, vulnerable population, lung-gut axis

## Abstract

Microbial communities form an important symbiotic ecosystem within humans and have direct effects on health and well-being. Numerous exogenous factors including airborne triggers, diet, and drugs impact these established, but fragile communities across the human lifespan. Crosstalk between the mucosal microbiota and the immune system as well as the gut-lung axis have direct correlations to immune bias that may promote chronic diseases like asthma. Asthma initiation and pathogenesis are multifaceted and complex with input from genetic, epigenetic, and environmental components. In this review, we summarize and discuss the role of the airway microbiome in asthma, and how the environment, diet and therapeutics impact this low biomass community of microorganisms. We also focus this review on the pediatric and Black populations as high-risk groups requiring special attention, emphasizing that the whole patient must be considered during treatment. Although new culture-independent techniques have been developed and are more accessible to researchers, the exact contribution the airway microbiome makes in asthma pathogenesis is not well understood. Understanding how the airway microbiome, as a living entity in the respiratory tract, participates in lung immunity during the development and progression of asthma may lead to critical new treatments for asthma, including population-targeted interventions, or even more effective administration of currently available therapeutics.

## Graphical Abstract

**Figure:**
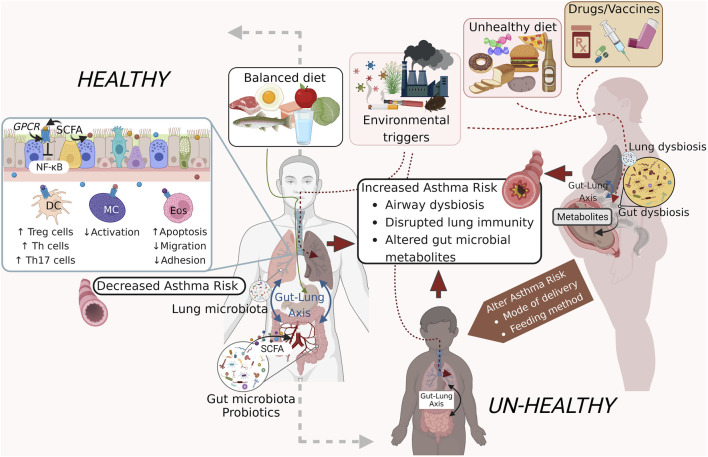
Exogenous factors that influence the microbiome and asthma pathogenesis. External factors including a well-balanced diet and clean air support a healthy gut microbiome that releases metabolites and short-chain fatty acids (SCFA) that promote a healthy pulmonary immune system that remains non-inflammatory thereby protecting against the development of asthma or asthma exacerbations (left panel). An unhealthy diet, dirty air, and some pharmaceutical agents on the other hand can induce dysbiosis in the gut and lung microbial communities through induction of co-morbid states like obesity and aberrant immune activation. Additional confounders including modes of delivery and feeding, use of antibiotics during pregnancy and childhood also have an impact on the airway microbial homeostasis and asthma onset and pathogenesis (right panel). Figure prepared on BioRender.

## 1 Introduction

The respiratory system, a member of the mucosal organ systems, is structurally and functionally complex with its own barrier and immune defenses. The large surface area, the continual availability of airway surface fluids, and perhaps constant ‘seeding’ from the ambient environment and microaspiration from the oro-gastro-intestinal system, establishes and maintains the respiratory system microbiome (Man et al., 2017) that begins colonizing shortly after birth (Senn et al., 2020). The basic definition of the microbiome as the “characteristic microbial community occupying a reasonably well-defined habitat which has distinct physio-chemical properties” (Whipps et al., 1988) may be extended based on ecological, host/environmental interactions, and genomic/methodological details (Berg et al., 2020). It is now well established that microbiota occupying various niches in the body play important roles in maintaining physical and mental health (Lynch and Pedersen, 2016; Man et al., 2017; Rieder et al., 2017; Chen et al., 2018; Halverson and Alagiakrishnan, 2020; Jarbrink-Sehgal and Andreasson, 2020; El-Sayed et al., 2021). Early life exposure to microorganisms is essential to train and shape the immune system, while microbial colonization acts like a barrier conferring protection against environmental pathogens (Nino et al., 2021). Conversely, some bacteria, viruses and fungi contribute to the development and pathogenesis of diseases. While the current literature on the airway microbiome has not provided definitive conclusions, it is strongly suggestive that the respiratory microbiome plays a role in the pathogenesis and control of chronic lung diseases like asthma. Currently, much of the published microbiome research is focused on the gut, where sampling techniques and sample acquisition are standardized. We anticipate that respiratory microbiome research will soon follow with the advent of more streamlined airway sampling methods and hypothesis-driven study designs (Carney et al., 2020). The airway microbiome has defined roles in lung diseases like cystic fibrosis (Lynch and Bruce, 2013), cancer (Goto, 2020), idiopathic pulmonary fibrosis (Amati et al., 2022), chronic obstructive pulmonary syndrome (Budden et al., 2019), and asthma, the focus of this manuscript.

Asthma is a chronic inflammatory airway disease with burgeoning global prevalence affecting 262 million people of all ages and causing an estimated 180,000 deaths worldwide (Braman, 2006; Asthma Fact Sheet World Health Organization, 2022a). Symptoms include wheezing, chest tightness and shortness of breath, which can vary in frequency and intensity (Global Initiative for Asthma, 2021). It is currently the most common chronic disease among children (Asthma Fact Sheet World Health Organization, 2022b) with 6 million pediatric asthma patients in the United States alone [Centers for Disease Control and Prevention (CDC)]. While many long-held beliefs about racial differences in biology and disease originated from social constructs, rather than any clearly defined biological differences, these constructs remain an integral part of our society and the literature. There are clear disparities in asthma prevalence and outcomes when looking at diverse populations and it is important to further define whether there are biological differences, socioeconomic issues, or healthcare access issues resulting in these differences. In some cases, we cannot determine the cause with current information, only that the disparities exist, and future effort should be devoted to clarifying these issues. Asthma prevalence is racially disparate, affecting approximately 16% of Black children compared to 7% of White children [CDC]. Our University and Hospital are located in Memphis Tennessee, a city on top of the list of ‘the worst places to live in with allergies’ (Allergy Capitals, 2022), the third poorest city in the United States with approximately 35% of children living in poverty (Memphis Poverty Fact Sheet, 2021) that have inconsistent access to healthcare. Additionally, nearly 80% of asthma patients in Memphis are Black (Oyana et al., 2017). Many of our patients suffering with asthma are faced daily with socioeconomic challenges making effective treatment of their disease more difficult. As such, we are deeply aware of the importance of approaching patient care contextually, addressing both causes of asthma as well as barriers to patient care and therapeutics.

There is no cure for asthma, and the choice for treatment management is challenging due to its immunological heterogeneity (Wangberg and Woessner, 2021). Asthma is categorized into two major endotypes; type 2 (T2)-high (eosinophilic) and T2-low (non-eosinophilic) based on their inflammatory profile (Kuruvilla et al., 2019). Although asthma etiology remains unknown, multiple genetic and environmental risk factors have been identified, including genetic and epigenetic variations, early severe viral infections, airborne environmental allergens (Mims, 2015) and particulates (Manisalidis et al., 2020), atopy, diet and nutrition, among others (Mims, 2015). Racial and socioeconomic factors add additional layers of complexity to asthma underscoring the importance of personalized medicine and a holistic approach to personalized treatment. Unfortunately, despite known increased prevalence of asthma, far fewer reports on asthma are focused on the Black population. For example, a PubMed search for ‘asthma children’ resulted in 64,072 hits while a search for ‘asthma children and Black’ only resulted in 1,414 hits (2.20%). Adding on the search term ‘microbiome’ only gave 3 hits, further emphasizing how little attention is paid to understanding apparent racial disparities in the literature. The aim of this review is to summarize and discuss the relationship between the airway microbiome and asthma pathogenesis in the context of extrinsic factors (environmental triggers, diet, medications, and mode of delivery) as well as intrinsic factors (genetics and the gut-lung axis) with a focus on the Black population as an understudied population across the country (Graphical Abstract). Literature on the respiratory tract (RT) clearly demonstrates that the airway microbiome varies by sampling location where the upper RT contains the highest microbial density (Charlson et al., 2011; Beck et al., 2012). As we focus on the impact external factors may have on the airway microbiome, we do not sub-stratify the airway microbiome by location within the RT. However, the reader is referred to a comprehensive review by Abdel-Aziz *et al.* for details on the airway microbiome by sampling location within the RT in asthma (Abdel-Aziz et al., 2019).

## 2 Airway microbiome in asthma

Approximately 15 years ago, the healthy human airways were shown to harbor a microbiome that remains poorly understood today, largely due to technical difficulties in access without contamination (Abdel-Aziz et al., 2019). The development of culture-independent molecular techniques such as the 16S rRNA sequencing have allowed researchers a detailed view of the microbial genome that has rapidly led to a bloom in the microbiome literature over the past decade. Although studies evaluating the respiratory microbiome are numerous, their significance is obscured by heterogeneity in study design including respiratory niche sampled and patient demographics among other variables (Abdel-Aziz et al., 2019; Carney et al., 2020). Microbial exposures/infections during human lung development are also of fundamental importance to the composition of the airway microbiome as rigorously reviewed by Mthembu *et al.* (Mthembu et al., 2021). As asthma exacerbations lead to muco-immuno-physiologic changes in the airways and lungs, the time at which the airway microbiome is sampled is of importance to capture waxing and waning communities that may correlate with disease severity. Furthermore, there may be significant differences in airway microbiome in asthma patients of different ethnicities or racial groups, and while these differences may be multifactorial or related to socioeconomic status, they may be impactful in disease progression and require personalized therapeutic approaches. Despite discrepancies that confound seamless comparisons between studies however, the existing literature on the airway microbiome has already made significant contributions to the field, compellingly demonstrating differences in microbial diversity and composition between asthma patients and healthy subjects. Although the microbiome is comprised of bacteria, viruses, fungi, archea, and parasites, here we focus on the dominant groups in the airway microbiome ecosystem and their interactions with the environment and host. A microbiome of high density and low diversity is positively associated with inflammation (Segal et al., 2014), therefore, in general, one would expect a break from the healthy airway microbiome which is highly diverse but lower in mass to correlate with the development of asthma in which airway inflammation is a hallmark.

### 2.1 Airway bacteriome

Bacteria are historically implicated in asthma pathogenesis and exacerbations. Culture-dependent studies have demonstrated the impact some species such as *Streptococcus pneumoniae*, *Haemophilus influenzae*, *Moraxella catarrhalis*, *Chlamydia pneumoniae*, and *Mycoplasma pneumoniae* may have on asthma onset, exacerbations, severity and response to therapeutics (Flores-Torres and Samarasinghe, 2022). A detailed culture-independent study published over a decade ago comparing airway microbiota from patients with chronic lung disease to healthy controls showcased the presence of an airway bacteriome that was characteristic of asthma (Hilty et al., 2010).

There is a general agreement that patients with asthma have a higher abundance of the phylum Proteobacteria in comparison with healthy controls in both the upper (Lee et al., 2019) and lower RT (Hilty et al., 2010; Huang et al., 2011; Marri et al., 2013; Zhang et al., 2016), although not all studies have found significant differences at the phylum level (Li et al., 2017). The phylum Proteobacteria includes a wide repertoire of potentially pathogenic bacteria, some of which have been found in higher abundance in asthma patients including the genus *Haemophilus* (Hilty et al., 2010; Boutin et al., 2017; Durack et al., 2017), *Moraxella* (Depner et al., 2017; Liu et al., 2020) and *Neisseria* (Durack et al., 2017; Sverrild et al., 2017). In contrast, healthy individuals have an abundance of the phyla Bacteroidetes and Fusobacteria (Pang et al., 2019), with *Prevotella* and *Veillonella* as the more common genera (Hilty et al., 2010; Denner et al., 2016; Zhang et al., 2016). Analyses of nasopharyngeal swabs from young and elderly asthma patients identified specific bacterial genes that associate with lung inflammation in addition to showing that differing members of the microbiome correlate with predicted FEV_1_ (%) in adults *versus* the elderly patients (Lee et al., 2019).

Asthma is a complex disorder with inflammatory signatures that vary by phenotype and differences in their respective microbiomes are to be expected. Neutrophilic asthma for example has lower bacterial diversity (Simpson et al., 2016; Taylor et al., 2018; Yang et al., 2018; Pang et al., 2019), richness (Simpson et al., 2016; Taylor et al., 2018; Yang et al., 2018; Pang et al., 2019), and evenness (Simpson et al., 2016; Taylor et al., 2018; Pang et al., 2019) and an increased abundance of Proteobacteria, especially *Haemophilus* and *Moraxella* (Taylor et al., 2018; Yang et al., 2018) compared to eosinophilic asthma patients. Furthermore, *M. catarrhalis* or *Haemophilus* colonization is positively correlated with sputum IL-8 and neutrophilia, longer asthma symptom duration, and lower FEV_1_ (Green et al., 2014). Experimental *in vivo* studies have shown that *H. influenzae* type b, non-typeable *H. influenzae* and *M. catarrhalis*, but not commensal *Prevotella* species, induce higher levels of pro-inflammatory cytokines (IL-8 and TNF-α) and neutrophil recruitment without eosinophil recruitment (Larsen et al., 2015). In contrast, patients with eosinophilic asthma may have a distinct microbiome with an increased abundance of the family Enterobacteriaceae (Li et al., 2017) and the genus *Tropheryma* (Simpson et al., 2016) and *Streptococcus* (Zhang et al., 2016). *S. pneumoniae* is particularly pertinent in asthma due to its increased carriage among asthma patients and possible association with exacerbations (Flores-Torres and Samarasinghe, 2022) therefore, increasing the likelihood that endogenous alterations in the airway bacteriome may occur during *Streptococcus* infections in asthma patients. The airway microbiome further differs based on the number of eosinophils in the airways. For example, patients with low eosinophil levels have an increased abundance of *Neisseria*, Bacteroidetes Actinobacteria, and decreased abundance of *Sphingomonas*, *Halomonas* and *Aeribacillus* species compared with patients with high levels of eosinophils (Sverrild et al., 2017). Generally, the bronchial bacterial burden is lower in T2-high asthma patients compared to T2-low asthma patients (Durack et al., 2017), potentially owing to diverse antibacterial functions of eosinophils (LeMessurier and Samarasinghe, 2019).

### 2.2 Airway virome

The human virome comprises all viruses that populate humans, including viruses that infect both eukaryotic and prokaryotic cells, and those integrated into the human genome (Wylie et al., 2012). Relatively little is known about the airway virome mostly due to technical challenges and fewer investigators in the field (Wylie et al., 2012). However, multiple diseases have been associated with virome dysbiosis including Crohn’s disease, type 1 diabetes, and obesity (Bai et al., 2022). The most commonly detected commensal viruses in children hospitalized with acute asthma exacerbations are rhinovirus C, bocavirus-1 and respiratory syncytial virus-B (Romero-Espinoza et al., 2018). Asthma patients have reduced abundance and diversity of the bacteriophage populations while the eukaryotic virome is increased predominantly by anelloviruses and picornaviruses (Megremis et al., 2020). The reduction in the phageome may be important as bacteriophages can control microbial populations, and the use of bacteriophages as a therapeutic strategy to reduce bacteria-induced asthma exacerbations has been proposed (Tzani-Tzanopoulou et al., 2021). While provocative, the use of phage-therapy must be approached with caution in asthma patients as bacteriophages are capable of natural transmission of antibiotic-resistant genes amongst member of the commensal microbiome (Muniesa et al., 2013; Colavecchio et al., 2017). A recent analysis of the sputum lung virome showed that severe asthma patients have an increased virome density dominated by β- and γ-herpesviruses that correlated with poor lung function compared to healthy controls (Choi et al., 2021). Studies on the airway virome in specific niches of the RT, in addition to enrollment of patients with different subtypes of asthma and varying demographics in parallel with the bacteriome would augment the field with clinically relevant information beneficial for future mechanistic studies.

### 2.3 Airway mycobiome

Environmental fungi are commonly allergenic to patients with asthma. In fact, severe asthma with fungal sensitization is a prevalent subcategory of asthma that is often eosinophilic and difficult to treat (Moss, 2014). Similar to the bacteriome and the virome, asthma patients have significant fluctuations in the airway mycobiome (van Tilburg Bernardes et al., 2020a). In contrast to the bacteriome however, bronchoalveolar lavage (BAL) of asthma patients have a higher fungal diversity (Liu et al., 2020) and relative abundance with predominance in *Trichoderma*, *Alternaria*, *Cladosporium*, and *Fusarium* compared to healthy individuals that have *Blumeria* and *Mycosphaerella* enrichment (Sharma et al., 2019). *Rhodosporidium* and *Pneumocystis* are abundant in BAL samples of pediatric patients with severe asthma although environmentally dominant *Aspergillus*/*Alternaria* species were not enriched (Goldman et al., 2018). Higher abundance of *Malassezia pachydermatis* (van Woerden et al., 2013) and *Cryptococcus pseudolongus* (Rick et al., 2020) have also been reported in the sputum of asthma patients in comparison with healthy cohorts.

Differences in mycobiome have been classified by asthma endotypes as well where T2-high asthma patients have a reduced fungal diversity (correlated with increased eosinophil numbers (Liu et al., 2020)) compared with T2-low patients (Sharma et al., 2019; Yang et al., 2022). Endobronchial brushings and BAL samples from T2-high adult asthma patients showed enrichment by *Alternaria, Aspergillus, Fusarium,* and *Cladosporium* and these taxa are suggested to serve as biomarkers of asthma phenotypes (Sharma et al., 2019). As with other microbes, the immune system is programmed to recognize and keep commensal fungi in check to prevent a ‘super bloom’. Environmental fungi are able to increase asthma susceptibility through a variety of antigens (chitin, β-glucan, proteases, etc.), that stimulate a T_H_2/T_H_17 bias (Tiwary and Samarasinghe, 2021). Then, perhaps dysbiosis in the mycobiome may promote asthma development through pulmonary immune stimulation (van Tilburg Bernardes et al., 2020a). Whether asthma patients with altered mycobiota are concurrently sensitized to environmental fungi, whether the mycobiome blooms after an environmental fungal exposure, and whether environmental fungi interact with commensal fungi in airways remain enigmatic and in need of investigation.

## 3 Airway dysbiosis in children and asthma development

The window between infancy and early childhood is crucial for immune and microbiome development through crosstalk at multiple levels (Zheng et al., 2020). Early-life also offers a critical window during which a signature-microbiome is imprinted wherein colonization may begin immediately after birth (Bosch et al., 2016; Chu et al., 2017) and lower airway microbiome matures in humans within the first two postnatal months (Pattaroni et al., 2018). The early-life airway microbiome is a critical regulator in immune programming to impede allergic asthma. The development of lymphoid tissues in the lungs are promoted by endotoxins early in life (but not in adulthood) (Rangel-Moreno et al., 2011). In mouse models of allergic asthma, the absence of microbial colonization (by maintenance in germ-free conditions or by administration of antibiotics early in life) increases total IgE and T2-associated cytokines in BAL, airway eosinophilia, and airway resistance (Herbst et al., 2011; Olszak et al., 2012; Russell et al., 2012). The shift in predominance from Firmicutes and Gammaproteobacteria to Bacteroidetes in early life induces a specific subset of regulatory T cells crucial for aeroallergen immune tolerance *via* PD-L1 in mice (Gollwitzer et al., 2014). Similarly, early-life fungal colonization also participates in host immune development and modulation during airway inflammation to ovalbumin (van Tilburg Bernardes et al., 2020b).

Colonization of the airway microbiome occurs after birth even in preterm infants. While bacterial DNA is detectable in tracheal aspirates of only a fraction of preterm infants during the first days after birth, all preterm infants have a measurable load of bacterial DNA by the first week of life (Mourani et al., 2011; Lohmann et al., 2014) thus exemplifying that the airway colonization occurs only after birth. Variations in the airway microbiome based on gestation time (term *versus* preterm) have been investigated and demonstrated that severely preterm infants who showed consistent dysbiosis of the airway microbiome after birth went on to develop bronchopulmonary dysplasia (Lal et al., 2016). Moreover, neonates with chorioamnionitis have reduced levels of *Lactobacillus* at birth (Lal et al., 2016). Whether airway colonization begins before birth remains contentious, the neonatal airway colonization is likely established early after birth and probably plays a significant role in the development of the long-term mucosal immunity.

The mode of delivery has a clear influence on asthma pathogenesis, as many studies have found that Caesarean section (CS) is a risk factor for asthma development (Thavagnanam et al., 2008; Guibas et al., 2013). The mode of delivery has a significant impact n the mucosal microbiota as well. Delivery by CS delays intestinal (Kim et al., 2020) and respiratory bacterial colonization, particularly reducing commensals related to health such as *Dolosigranulum* and *Corynebacterium* (Bosch et al., 2016). In addition, infants born through vaginal delivery have higher bacterial load in the lower airways, particularly with more Firmicutes and *Lactobacillus* species compared to CS delivery (Cardelli et al., 2021). History of CS is a risk factor for future hospitalizations due to viral infections during infancy and childhood (Moore et al., 2012; Kristensen et al., 2015; Miller et al., 2020; Alterman et al., 2021), which, depending on the infectious agent, may increase the risk of asthma onset (Gern, 2000; Beigelman and Bacharier, 2016; Flores-Torres and Samarasinghe, 2022). Correspondingly, children delivered by CS have a higher risk of RSV-induced hospitalization, subsequent wheezing, and asthma symptomatology compared to those delivered vaginally (Zomer-Kooijker et al., 2014; Kristensen et al., 2015). Neonatal gut *Clostridium* abundance in early life after CS delivery (Shaterian et al., 2021), may promote asthma risk in later life (Penders et al., 2007). This is concerning, as the number of CS performed (sometimes performed as a patient preference) is increasing globally, with projections that nearly a third of all births will be by CS by 2030 (WHO, 2021). Black women have been found to be more likely to have a CS compared to other races in the US (Edmonds et al., 2013; Valdes, 2021), with increased incidence from 22 to 64% (Valdes, 2021). The link between increased incidence of asthma and CS delivery suggest that this is an area for intervention and that research efforts should be focused on understanding the drivers for increased CS. Longitudinal analyses of the gut microbiome in CS delivered *versus* vaginally delivered infants with emphasis on maternal demographics may help elucidate the long-term impact CS may have on the health of the baby.

## 4 Lung-Gut axis

The epithelia of the respiratory and gastrointestinal tracts share a common embryonic origin thus sharing mucosal functions despite performing physiologically distinct roles (Chunxi et al., 2020). The microbiota of the GI tract is the highest in abundance in the human body at 10^14^ bacteria in comparison with the low abundance in the lower RT of 10–100 bacteria/1,000 human cells (Marsland et al., 2015). *Bifidobacterium*, *Faecalibacterium*, and *Bacteroides* represent the most abundant genera in intestinal microbiota (Marsland et al., 2015) encompassing clusters of bacteria known as enterotypes. Bifidobacterium species in the gut are important inducers of antimicrobial peptides and immunoglobulin A (IgA) production that are vital for broader mucosal protection (Pilette et al., 2004; Mantis et al., 2011; Budden et al., 2017; Budden et al., 2019).

Metabolites of gut microbiota regulate the tone and composition of the gut microbiota communities as well as immunological responses capable of affecting distal organs including the lungs as reviewed previously (Stavropoulou et al., 2020). Variations in the makeup and abundance in the gut microbiome have been associated with lung diseases and it is estimated that microbiota of each organ may cross-regulate during health and disease (Anand and Mande, 2018; Barcik et al., 2020). Since the immune system is educated by non-self-patterns, it is no surprise that the gut-lung microbiota axis can program and regulate the immune system. Gut metabolites such as short-chain fatty acids (SCFA) serve either as an energy source, or as signaling molecules (Anand and Mande, 2018), by binding to G-protein coupled receptors initiating host immune cascades (Sun et al., 2017). Microbiome-secreted SCFA hinders the development of allergic asthma *via* several mechanisms including signaling through G-protein coupled receptor 41 (Sun et al., 2017), inhibition of histone deacetylases (Verstegen et al., 2021), and immunoregulation of dendritic cells (DCs), T and B lymphocytes to be non-reactive to environmental allergens in experimental models of allergic asthma (Trompette et al., 2014; Kim et al., 2016; Cait et al., 2018; Dang and Marsland, 2019). During diseases that weaken mucosal barrier integrity such as sepsis and acute respiratory distress syndrome, there may be opportunities for the direct transfer of microbiota across the gut-lung axis (Budden et al., 2017). Indeed, dysbiosis in neonatal gut microbiota with depleted *Bifidobacteriaceae*, *Lactobacillaceae*, *Clostridiceae* bacteria and enriched *Candida* and *Rhodotorula* fungi together with metabolites such as lipokines and sterols that inhibit regulatory T cells are associated with atopy and allergy development in childhood (Fujimura et al., 2016).

The “common mucosal response” theory is gaining popularity with increasing evidence suggesting that effects of the microbiome on gut mucosal immunity may influence mucosal immune responses in the lungs (Anand and Mande, 2018). Mucosa-associated lymphoid tissue (MALT), the main component of the mucosal immune system, includes the gut-associated lymphoid tissues (GALT) and the bronchus-associated lymphoid tissues (BALT), both of which perform situational functions as inducer and/or effector sites (Anand and Mande, 2018; Barcik et al., 2020). Microfold cells that line the GALT take up gut microbial antigens transferring them to DCs in subepithelial regions (Anand and Mande, 2018) to initiate T and B cell responses including IgA production (Anand and Mande, 2018). Conversely, gut dysbiosis can alter the balance between immunoglobulins to promote IgE levels either through increasing sensitivity to allergens or degrading IgA cumulatively leading to atopy/allergy (Fujimura et al., 2016; Salameh et al., 2020). Thus, gut commensals act as important immune informers, especially in asthma pathophysiology.

Differences in gut microbiota have been associated with disease susceptibilities by race and sex. Although minimal emphasis is placed on microbiome profiling by race, analysis of the gut microbiome may be a simple and early biomarker for disease predisposition provided sufficient information is made available in health and disease states across racial groups. For example, while the composition and abundance of Firmicutes and Bacteroidetes were equivalent between Black and White populations, Actinobacteria and Verrucomicrobia were more abundant phyla in Black women particularly in relation to insulin resistance compared to White women (Price et al., 2022). This difference suggests that a linkage exists between microbiome and race in the context of disease susceptibility and severity (Brooks et al., 2018). While these susceptibilities may be the result of socioeconomic differences rather than inherent biological differences, recognizing and understanding the origins of these differences may be valuable determining the appropriate interventions.

## 5 Factors that affect the airway microbiome

### 5.1 Genetics

Host genetics also govern the airway microbiome composition based on genome-wide association study (GWAS). Host genes involved in shaping the airway microbiota include those important in pattern recognition receptors, barrier defense and mucosal immunity, MHC/HLA specificity, cytokine and chemokine signaling including the JAK/signal transducer and activator of transcription pathway, and vitamin D signaling (Tang et al., 2021). GWAS correlating host genetics and airway microbiota show positive association between mucosal immunity genes (such as *FUT3*, *PGLYRP3*, and *PGLYRP4*) and the relative abundance of upper RT microbiome (Igartua et al., 2017).

The fact that close relatives have significantly reduced beta-diversity in the airway microbiome further implicates the importance of host genetics on the make-up of the airway microbiome (Igartua et al., 2017). Data on host genetic variations in 93 individuals and respective bacterial abundance retrieved from the Human Microbiome Project, showed significant association between host genetic variation and composition of microbiome in upper RT components anterior nares, throat, and tongue dorsum (Blekhman et al., 2015). These authors performed further analysis to elucidate the biological processes involved in host genetics and microbiome interaction and found substantial enrichment within the leptin signaling pathway suggesting that leptin may regulate the microbiome (Blekhman et al., 2015). Leptin, the protein derivative of the obesity gene, is a significant biomarker of asthma in children (Sood et al., 2006).

Innate immune signaling cascades are increasingly gaining traction for their role in asthma pathogenesis (Zakeri and Russo, 2018) and creating the setting for the airway microbiome (Lipinski et al., 2021), so much so that pattern recognition receptor manipulation is a successful therapeutic avenue for asthma (Bezemer et al., 2012). Polymorphisms in *CD14* rs2569190 and *TLR2* rs13150331 in asthma patients influences the microbial composition (Losol et al., 2021). Asthma patients with genotypes AG/GG at *CD14* rs2569190 showed elevated levels of neutrophils and lower levels of *Prevotella* and *Dolosigranulum* compared to those with the AA genotype (Losol et al., 2021).

### 5.2 Smoking

Smoking is a major cause of several chronic airway diseases such as chronic obstructive pulmonary disease (COPD), lung cancer (Cox et al., 2011) and asthma (Huang and Shi, 2019) wherein smokers have more severe symptoms compared to non-smokers and smoking cessation drastically improves disease control (Munck et al., 2016). Since the airway microbiome plays a crucial role in disease pathology, several studies have attempted to elucidate the effect of smoking on the airway microbiome. Of the 4,000 chemical compounds found in tobacco smoke, about 100 have known hazards to human health (Pietinalho et al., 2009). However, how specific compounds within cigarette smoke may impact the respiratory microbiome is notably under investigated.

As expected, smokers’ microbiome differed between the upper and lower RTs with no significant difference in the relative abundance of specific microbes of lower RT (Morris et al., 2013). Compared to the oral microbiome of non-smokers, the relative abundance of species belonging to *Porphyromonas*, and *Gemella* was substantially less in smokers (Morris et al., 2013). Although electronic cigarettes (EC) have been introduced as a relatively low-risk alternative to tobacco cigarettes, studies have only recently started to address the paucity of data concerning adverse effects of EC use on respiratory health including the airway microbiome (Chopyk et al., 2021). In comparison to non-smokers/-vapers, EC users have greater alpha diversity in saliva samples, and a substantial shift in beta diversity in buccal mucosa samples (Chopyk et al., 2021). A significant increase in the abundance of bacteria such as *Veillonella* and *Haemophilus* was also observed in EC users together with increased nasal colonization by *Staphylococcus aureus* (Chopyk et al., 2021). The oral microbiome has been compared to the lung microbiome in cigarette smokers, EC users, and never-smokers (Ying et al., 2022). The lung microbiome in cigarette smokers differs from that of EC users and non-smokers, where most bacteria including *N. elongata*, *N. sicca*, and *H. parainfluenzae* are decreased in smokers. Comparing the oral microbiome of smokers *versus* never-smokers, and smokers *versus* EC users showed differential abundance of 152 species and 17 species, respectively, but only 21 species differed in their abundance within the lung and oral cavity (Ying et al., 2022).

The airway microbiome in the context of smoking show increased relative abundance of Firmicutes, Actinobacteria, and *Propionibacterium* species and a decrease in Betaproteobacteria abundance in smokers (Pfeiffer et al., 2022). A positive correlation was observed between lung specific Bacteroidetes and long-term smoking compared to the negative correlation observed between *Prevotella* and *Veillonella* genera smoking years (Pfeiffer et al., 2022). The relative abundance of upper RT *Leptotrichia* and *Centipeda* increased and associated with the levels of nicotine metabolites and smoking frequency (Pfeiffer et al., 2022).

The depletion of oxygen in oropharynx can lead to the outgrowth of anaerobes and facultative anaerobes like *Streptococcus* and *Veillonella* although commensals like *S. aureus* are not altered by smoking status (Pfeiffer et al., 2022). The symbiotic relationships of several bacteria in upper and lower airway can be disrupted by smoking, thus affecting lung health. Compared to never-smokers, smokers have a lower relative abundance of Proteobacteria in the upper airways and higher abundance in the lungs. This could be due to epithelial cell injury caused by cigarette smoke (Spira et al., 2004) that permits colonization by biofilm-forming bacteria (Invernizzi et al., 2020). Pfeiffer *et al.* reported that such biofilm-forming bacteria included Betaproteobacteria and Gammaproteobacteria, and were detected solely in the lungs of smokers (Pfeiffer et al., 2022). Striking similarities in the abundance of airway microbiome of smokers *versus* non-smokers were reported by Charlson *et al.* (Charlson et al., 2010) wherein the anaerobic Firmicute *Megasphaera* species significantly increases alongside increased abundance of potential pathogens like *Actinomyces*, *Atopobium*, *Streptococcus*, and *Veillonella* species. Significant increase in the abundance of *Haemophilus* has also been observed in smokers’ sputum compared to non-smokers (Wang et al., 2019). A recent study on a Chinese cohort found an increase in the relative abundance of the genera *Haemophilus*, *Rothia, Neisseria, Actinomycetes, Porphyrins, Streptococcus*, and *Acinetobacter* in smokers was observed (Liu et al., 2022).

Dysbiosis and alterations in relationships within microbial communities that result from smoking may depend on smoking mode, frequency, ingredients, and underlying co-morbidities. For instance, microbes such as *Pseudomonas aeruginosa*, *Acinetobacter*, *Clostridium*, *Bacillus*, *Burkholderia*, and *Klebsiella* have been detected in cigarettes made in European Union (Huang and Shi, 2019). Smoking also affects the respiratory epithelial barrier and function which cascades to alterations in the pulmonary immune response including antimicrobial defense strategies that may keep the airway microbiome in check. Importantly, the change in the microenvironment of the airway due to smoking such as reduced oxygen, pH shift, and acid production may also aid in the outgrowth of microbes that are anaerobic (Huang and Shi, 2019). While smokers who have asthma show a greater bacterial diversity in comparison to non-smokers, smoke cessation does not alter bacterial diversity (Munck et al., 2016). Thirdhand smoke exposure in children has also been reported to cause an alteration in their airway microbiome, in a manner similar to studies reported earlier for smokers, which includes changes in the abundance of *Streptococcus*, *Corynebacterium*, and *Staphylococcus* (Kelley et al., 2021).

Glycerol, propylene glycol and several flavorings are key chemical ingredients in ECs which may or may not consist of nicotine (Farsalinos et al., 2014). Although studies suggest that ECs may only release low levels of toxic compounds, the use of food-approved flavorings in EC may pose a health risk upon inhalation (Farsalinos et al., 2014). For instance, a study focusing on the effect of flavored EC, with or without nicotine, showed a substantial negative effect on pathophysiology of allergic airways disease in mice (Chapman et al., 2019) and occupational inhalation of food flavorings has been known to aggravate asthma symptoms (Clapp and Jaspers, 2017). Thermal decomposition of EC liquid ingredients propylene glycol and vegetable glycerin, pose a risk of production of reactive carbonyl compounds such as acrolein, formaldehyde, and acetaldehyde which have known toxic properties (Clapp and Jaspers, 2017). Moreover, rodent models have revealed that nicotine exposure of dams causes lung developmental abnormalities in the offspring, and lead to transmission of asthma-like symptoms through multiple generations (Clapp and Jaspers, 2017) suggestive of epigenetic imprinting. As ECs can have a plethora of effects on the pulmonary system including the microbiome, detailed studies on the effect of EC on respiratory microbiome in asthmatics and non-asthmatics would be pertinent (Hickman and Jaspers, 2020).

The percentage of the White and Black populations that smoke is comparable (Smoking & Tobacco use, 2022), however, intriguingly, Black smokers suffer from more significant negative consequences of smoking (Mendez and Le TTT, 2021) including asthma (Marchese et al., 2015) than their White counterparts. Factors including the age of smoking onset, smoking index, pack years, housing type (shared ventilation systems vs. single), and second-hand smoke exposure require further investigation to better understand the impact smoking has on the pediatric population in correlation with asthma symptoms and may contribute to the race-associated differences reported between White and Black smokers.

### 5.3 Diet and nutrition and other socioeconomic factors

The co-occurrence of asthma and obesity is well-recognized as associative, although mechanistic insights are not fully elucidated. Obesity is a risk factor not only in adults, but also in children, and the increase in the risk of asthma development due to obesity may start *in utero* (Dixon and Que, 2022). Furthermore, the occurrence of maternal obesity is independently associated with a 15–30% increase in the risk of asthma in the progeny although this risk is also governed by inflammatory mechanisms and pre-/post-natal changes (Dixon and Que, 2022). The role of obesity-associated inflammatory response which can ultimately lead to asthma, and the contributory role diet plays in this relationship has been validated (Calcaterra et al., 2021). In obese patients with asthma, food components such as sugar, fat, and inferior-quality nutrients have been associated with the inflammatory response (Calcaterra et al., 2021). The US Department of Agriculture has identified four zip codes within the city of Memphis as being food deserts, where families do not have access to grocery stores (Economic Research Service, 2022). Children living in such environments regularly consume a diet high in fat, low in fiber, and low in methylation, all of which promote asthma severity and airway dysbiosis (Montrose et al., 2017; Rosa and Perzanowski, 2019; Musso et al., 2020; Toivonen et al., 2020; Wen et al., 2022).

Breastmilk is a rich admixture of macro- (protein, fat, and lactose) and micro- (vitamins, trace elements) nutrients, bio-actives (hormones, antibodies, cytokines, antimicrobials, and exosomes) (Ballard and Morrow, 2013). Wasilewska *et al.* showed a strong correlation between duration of breastfeeding and health status of children wherein the breast-fed group of infants had comparatively lower incidence of hypersensitivity to allergens and subsequent asthma development (Wasilewska et al., 2022). While the source remains controversial, breastmilk also contains a microbiome abundant in *Lactobacillus* and *Bifidobacterium* species that are likely to promote infant gut health (Ruiz et al., 2019). Compared to breast-fed babies, formula-fed babies show a lower bacterial-diversity and are more prone to developing asthma. For example, maternal TGF-β and IgA are crucial for intestinal homeostasis (Oddy, 2017) and protection against obesity and allergic disease later in life (Oddy, 2017). While the benefits of breast-feeding are well accepted (Wang et al., 2017) including the mitigation of potentially detrimental effects of antibiotic use during pregnancy (Huo et al., 2018), breastmilk itself may also be detrimental as a source of obesogenic factors, as opposed to protective components (Isganaitis, 2021).

The link between diet and microbiota is evident from the co-occurrence of gut dysbiosis and malnutrition. A diet that is rich in nutrients and dietary fibers can result in a diverse gut microbiome that is favorable for host immunity and health (Anand and Mande, 2018). A compromised diet not only affect the intestinal health, but also leads to chronic pulmonary disorders such as COPD and asthma (Anand and Mande, 2018). The impact of variation in diet and dietary components on the composition of microbiota of the host has been reviewed extensively (Singh et al., 2017; Tomova et al., 2019; Zhao et al., 2019; Peled, 2021; Wu et al., 2022). However, the modes by which the microbiota composition is altered, either due to interactions between dietary components and the microbiota, or due to crosstalk between microbes themselves as a result of the variation in diet, remains less clear.

#### 5.3.1 Carbohydrates

Gut microbiota utilize complex carbohydrates and soluble fibers to produce metabolites including SCFA by anaerobic fermentation. These SCFA in turn play a potential role in maintaining a “normal” gut microbiota. Considering this, and the role of a normal microbiome in maintaining pulmonary health, the contribution of dietary carbohydrates and fibers to gut-lung health is significant. SCFA produced by gut microbiota contains acetate, propionate, and butyrate at a 60:20:20 proportion (Machado et al., 2021) thereby making acetic acid, propionic acid, and butyric acid the most abundant SCFA in the human gut (Yip et al., 2021). In addition to serving as immunomodulators, SCFA may also either act as a carbon source for certain gut microbiota or be toxic (at higher concentrations) to other microbial community members (Sun and O'Riordan, 2013). SCFA production causes changes to the gut pH which has an important regulatory role on the gut microbiota composition (Krautkramer et al., 2021).

Several studies indicate that the risk of asthma is lower in subjects who consume a fiber-rich diet or have high levels of SCFA (Lee-Sarwar et al., 2020; Yip et al., 2021; Wen et al., 2022). Allergic asthma is ameliorated by butyrate *via* various mechanisms such as suppressing the activation of DC and migration to local lymph nodes, by inhibiting B cell isotype class switching and differentiation of plasma cells to reduce circulating IgE (Yip et al., 2021). Maternal SCFA supplementation during pregnancy in a mouse model reduced allergic airways disease development in the offspring (Lee-Sarwar et al., 2020). Similarly, infant fecal SCFA levels correlate with reduced asthma development as one year old infants with high fecal butyrate and propionate levels have 50% reduced sensitivity to allergens by age six (Yip et al., 2021). A correlation exists between the presence of butyrate or butyrate-producing commensals, and the severity of allergic asthma in mice as well as humans (Yip et al., 2021). Susceptibility towards allergic outcomes in children is higher in cases of low SCFA levels in early life (Cheng et al., 2022). SCFA show immunomodulation by increasing T regulatory cell differentiation (Lee-Sarwar et al., 2020), a cell population that seems important in alleviating allergic asthma in experimental models (Kearley et al., 2005; Lewkowich et al., 2005; Baru et al., 2010) although their specific role in human disease is still unclear (Robinson, 2004).

Most studies have associated high fiber diets with elevated SCFA production by gut commensals, health, and ultimate reduction in asthma occurrence. It is also possible that high fiber diets alter the microbiome without affecting SCFA production and subsequently reduce lung inflammation and asthma (Wen et al., 2022). Herein, the gut microbiome was altered by a new genus of lipid-metabolizing microbes such as *Romboutsia* and *Ruminococcus torques* that were enriched by a high-cellulose diet (Wen et al., 2022). Although not directly tested, positive associations between dietary fiber intake in asthma patients and improved lung function (Berthon et al., 2013) are likely through direct/indirect microbiome regulation. Both Black and White populations have reduced fecal SCFA content compared to Native Africans (O'Keefe et al., 2009), and incorporating fiber-rich food has led to significant alterations to the gut microbiome thereby improving mucosal health in Black cohorts (O'Keefe et al., 2015). Therefore, ensuring that Black children, especially, have access to fiber-rich food may promote gut microbiome health and reduce asthma pathogenesis.

#### 5.3.2 Proteins

The various states that proteins exist in the gut based on post-translational modifications, digestion, and their interaction with other food components, can all affect the composition of the gut microbiota (Wu et al., 2022). Furthermore, protein source also affects the gut microbiome; hen egg white-fed rats show relatively higher levels of *Akkermansia* while duck egg white-fed rats have higher abundance of *Peptostreptococcaceae* and *Proteobacteria* and lower abundance of *Lachnospiraceae* (Yu et al., 2020). On the other hand, soy proteins improve abundance of *Bifidobacteria* and *Lactobacilli* while decreasing Bacteroidetes (Huang et al., 2016; Ashaolu, 2020). Variations in nutritional protein source could play a vital role in asthma symptom control.

A study comparing children from an African village on an agrarian diet (rich in fiber and low in animal protein and fat) and children from the European Union on a Western diet (rich in fat and sugar) showed substantial differences in the gut microbiota. Children on the agrarian diet showed enrichment in Bacteroidetes, depletion in Firmicutes, higher SCFA content, and lower levels of *Escherichia* and *Shigella* (De Filippo et al., 2010). As expected, *Prevotella* and *Xylanibacter* (genetically equipped to digest fiber to produce SCFA) were more abundant in the African children (De Filippo et al., 2010). Thus, plant-based high protein diet could potentially help reduce asthma risk in all children. Surviving human biliary secretions that can be cytotoxic to prokaryotes requires microbial adaptation (Begley et al., 2005). Diets rich in animal protein led to abundance of bile-tolerant bacteria such as *Bacteroides*, *Alistipes*, and *Bilophila*, and lowered the levels of Firmicutes *Roseburia*, *Ruminococcus bromii*, and *Eubacterium rectale* (David et al., 2014). As a change in proportion of functionally distinct bacterial populations can occur when specific macronutrients are favored or limited, a diet that is disproportional can cause dysbiosis (Zhao et al., 2019). Indeed, a long-term high protein diet causes gut dysbiosis along with gut inflammation with heightened permeability, kidney damage, and elevated risk of developing metabolic (type 2 diabetes and obesity) and cardiovascular diseases (Snelson et al., 2021; Cai et al., 2022). However, the intake of a protein-rich diet can help reduce fat mass (Geiker et al., 2018) which could help improve asthma symptoms. A diet enriched with meat does not impact lung function in adults with asthma (Hooper et al., 2010) emphasizing that a single cause and effect relationship between macromolecules and asthma may not exist.

#### 5.3.3 Fats

A diet high in fat, does reduce FEV1 in adult asthma patients possibly through direct impact on inflammation (Wood et al., 2011), impacting obesity (Jessri et al., 2017), and the microbiome (Murphy et al., 2015). Several studies have reported on changes in the composition of the gut microbiota as a function of dietary fat (Fava et al., 2013; Costantini et al., 2017; Muralidharan et al., 2019; Wolters et al., 2019; Mokkala et al., 2020), however, very few of them have attempted to decipher the mode by which fats affect the microbiota composition. Studies on the effect of high-fat diet on the microbiota composition are contradictory likely because food is complex and considering the effect of a particular dietary component on the microbiota composition does not address the synergistic effect of other dietary components.

Gut dysbiosis from fat-rich diets, may be compounded by other dietary components. Nuts, for instance, are rich in unsaturated fatty acid, fiber, as well as various bioactive phenolics and minerals. Some of these components reach the colon in an intact form, ultimately affecting the composition of microbiota. Phenolics, and the SCFA such as butyric acid produced from nut fiber can positively alter gut microbiota to yield a healthier composition. Nuts may function as prebiotics either by increasing the abundance of Bifidobacteria or lactic acid bacteria (Muralidharan et al., 2019) or by providing an additional fat source due to incomplete absorption (Cassady et al., 2009).

In the case of planned diets such as the very low-calorie ketogenic diet (VLCKD), the consumption of fat is high (Alsharairi, 2020). Generally, a fat-rich diet can cause dysbiosis. However, dysbiosis from high-fat diet drastically differs from that of a VLCKD wherein *Bifidobacterium* species are reduced in abundance largely as ketone bodies directly hinder *Bifidobacterium* growth (Ang et al., 2020). VLCKD has been associated with altered gut microbiota in pediatric patients and reduced asthma risk, perhaps by metabolic reprogramming and epigenetic markers (Alsharairi, 2020) or reducing allergen-induced airway inflammation by inhibiting innate lymphoid cells in the lungs (Karagiannis et al., 2020). Supplementing the diet with polyunsaturated fatty acids like eicosapentaenoic acid and docosahexaenoic acid substantially decreases abundance of Bacteroidetes while increasing Firmicutes in mice (Mujico et al., 2013) potentially through the alteration of the intestinal wall fatty acid composition thus affecting gastrointestinal microbiota attachment (Costantini et al., 2017). Alternatively, fatty acids could get incorporated into bacterial cell membranes which also affects their adhesion properties (Mokkala et al., 2020), possibly favoring certain microbes over others. The metabolites produced by the gut microbiota as a result of high-fat diet may affect their composition either by modifying the toxicity locally of the intestinal environment, or by systematically inducing low-grade inflammation (Mokkala et al., 2020).

Diets rich in omega-6 fatty acids can impact metabolism and inflammation. Compared to mice fed with more omega-6 fatty acids, mice given more omega-3 fatty acids showed undetectable levels of LPS-producing pro-inflammatory bacteria, including the phyla Proteobacteria (gamma- and delta-proteobacteria, *E. coli*, *Fusobacterium*, *Prevotella*, segmented filamentous commensals and *Clostridium* cluster XI (Kaliannan et al., 2015). Mice that were given more omega-3 fatty acids also had elevated levels of bacterial groups such as *Lactobacillus* (primarily *L. gasseri*), *Bifidobacterium*, *Enterococcus faecium*, *Akkermansia muciniphila*, and *Clostridium* clusters IV and XIVa which are anti-inflammatory and/or LPS-suppressing (Kaliannan et al., 2015).

Antimicrobial properties of fatty acids may also exert a direct effect on microbiota composition. Ethyl ester of eicosapentaenoic acid inhibits the growth of anaerobe symbiont, *Bacteroides thetaiotaomicron*, a representative member of Bacteroidetes, whereas linoleic acid inhibits *Lactobacillus* strains (Mokkala et al., 2020). Conversely, medium chain fatty acids, which are abundant in breast milk, may increase the growth of *Lactobacillus* and *Bifidobacterium*, highlighting their role in early development of gut microbiota (Nejrup et al., 2015). It is worth noting that both, *Lactobacillus* and *Bifidobacterium* have been known to substantially alleviate asthma and other allergic conditions (Ozdemir, 2010; Liu et al., 2021). Aforementioned animal protein-rich diets are also accompanied by high fat content that promoted bile secretion. Intriguingly, the microbiota composition of mice fed with a bile-supplemented diet is similar to mice fed with high-fat diet (Zheng et al., 2017).

#### 5.3.4 Probiotics

The beneficial effects of probiotics include stimulation of anti-inflammatory cytokines, enhancing mucosal barrier function, and modification of the gut microbiome composition (Singh et al., 2017; Stavropoulou and Bezirtzoglou, 2020). Regular consumption of probiotic foods such as fermented milk and yogurt leads to an increase in bacterial load, especially beneficial gut bacteria such as Bifidobacteria and/or Lactobacilli (Singh et al., 2017) which play significant roles in asthma symptom alleviation (Ozdemir, 2010). Studies have also exemplified that probiotic bacteria aid in re-establishing normal microbiota after perturbation (Sanders, 2016). For instance, the use of antibiotics is associated with antibiotic-associated diarrhea typically caused by the outgrowth of *Clostridioides difficile*. The use of *Lactobacillus rhamnosus* GG and *Saccharomyces boulardii* after the termination of antibiotic treatment has been reported to control the outgrowth of *C. difficile* and restore the microbiota to normalcy (Ceapa et al., 2013).

The transient probiotic bacteria can modulate commensals by producing anti-microbial compounds such as reuterin (produced by *Lactobacillus reuteri*) and plantaricin (produced by *Lactobacillus plantarum*), or indirectly *via* immune system modulation or altering the gut barrier function (Ceapa et al., 2013). Probiotic microbes may also modulate outgrowth or abundance of specific microbiota such as *Roseburia intestinalis* or *Eubacterium halii*, which are lactate-using firmicutes capable of producing various kinds of SCFA (O'Keefe et al., 2009). Cumulatively, functions of probiotic bacteria may help regain a favorable microbiome after stress.

#### 5.3.5 Vitamins

Since vitamins are efficiently absorbed in the proximal small intestine, and do not tend to reach the distal gastrointestinal tract, they were not thought to affect the composition of gut microbiota. However, administration of higher doses to escape complete absorption (Mandal et al., 2016) and colon-targeted delivery (Fangmann et al., 2018) are efficacious modes of gut microbiome modulation (Pham et al., 2021). Each vitamin may aid in maintaining the pulmonary health *via* one or more mechanisms. Vitamins A, C, and E for instance, can alleviate asthma severity by downregulating oxidative stress which exacerbates airway inflammation (Han et al., 2013). Vitamin A may also act directly on the immune system by inhibiting T_H_2 and T_H_17 responses while vitamin C may reduce airway reactivity and inflammation through prostaglandin inhibition (Han et al., 2013). The dietary intake of vitamin A and vitamin C in asthma patients is significantly low (Allen et al., 2009), and children with severe asthma have low plasma vitamin A levels (Samarasinghe et al., 2020). In comparison to mild asthma, vitamin E levels are significantly low in severe asthma (Allen et al., 2009). Vitamin D also plays a crucial role in pulmonary health, and similar to children with eosinophilic esophagitis (Armbruster-Lee et al., 2018), vitamin D deficiency/insufficiency are associated with asthma pathogenesis (Wang et al., 2022). A strong correlation has been observed between vitamin D deficiency and asthma in Black children (Paul et al., 2012). Interestingly, other risk factors for developing asthma such as urban residency and obesity are also observed with vitamin D deficiency (Paul et al., 2012). Independent of racial ancestry, an association between vitamin D deficiency and asthma exacerbations has also been observed in Puerto Rican children (Brehm et al., 2012). Although the exact mode of action of vitamin D in asthma prevention is not well-understood, it may be through regulation of immune response or gene expression, enhancing responsiveness towards steroids, influencing lung development/function, prevention of weight gain, or by protecting against viral infections (Han et al., 2013; Armbruster-Lee et al., 2018).

Although humans depend greatly on their diet for vitamin acquisition, gut microbiota also serve as a source (Rowland et al., 2018; Pham et al., 2021). Vitamins (such as B vitamins) produced by the gut microbiota may primarily be utilized by microbes of symbiotic association, thus not being able to provide the host with the daily recommended intake (Magnusdottir et al., 2015). Markers of folate deficiency, for instance, have been associated with asthma as well as attacks of shortness of breath (Thuesen et al., 2010). Others such as vitamin K are predominantly produced by Firmicutes, Proteobacteria, and Bacteroidetes in the gut (Ravcheev and Thiele, 2016). Vitamin K is a crucial co-factor for periostin (Coutu et al., 2008) which is significantly increased in asthma patients (Jia et al., 2012), linking gut dysbiosis to lung disease.

#### 5.3.6 Minerals

Minerals can be divided into three groups based on their role in asthma pathophysiology: 1) minerals (such as copper, zinc, and selenium) that are involved directly in immunity, 2) minerals (such as chromium, iodine, iron, and manganese) that are involved in obesity, disturbances in thyroid gland, anemia, and oxidative stress, and 3) the minerals of minor or unknown significance in asthma (Zajac, 2021).

Commensals of the gut are sensitive to mineral levels, utilize them, and regulate availability for the host (Barone et al., 2022). The mechanism by which the minerals affect the microbiota composition is interesting. For instance, iron availability correlates with butyrate production by pediatric gut microbiota and iron deficiency leads to a reduction in butyrate-producing anaerobe bacteria such as *Roseburia* species, *E*. *rectale* and members of *Clostridium* cluster IV, such as *F*. *prausnitzii* (Dostal et al., 2015). The gut microbiota also plays a crucial role in improving the bioavailability of iron for the host. For instance, *p*-hydroxyphenyllactic acid secreted by the probiotic microbe *L. fermentum* reduces ferric iron to ferrous, thus making it bioavailable to the host (Gonzalez et al., 2017). Accordingly, intestinal epithelia of germ-free mice showed a 2-fold decrease in ferroprotein abundance, as well as iron deficiency, which was restored in the presence of the commensals *B. thetaiotaomicron* and *F. prausnitzii* and the probiotic bacterium *S. thermophilus* by a 12-fold induction of ferritin in colon (Deschemin et al., 2016). Asthma and poor lung function are associated with reduced serum iron levels possibly due to iron loading that occurs in leukocytes recruited into the lungs during exacerbations (Brigham et al., 2015; Ali et al., 2020; Zajac, 2021).

Gut microbiota also play a role in improving the bioavailability of other minerals. Lactobacillus strains such as *L. paracasei* LPC09 (DSM 24243) have been shown to degrade oxalates, which otherwise hinder the absorption of calcium in the host (Mogna et al., 2014). Fiber-rich diets enrich Bacteroidetes (*Parabacteroides*) and Firmicutes (*Clostridium*) that produce SCFA (Whisner et al., 2016) through calcium absorption. Additionally, SCFA-driven reduction in pH boosts calcium bioavailability to the host by hindering calcium phosphate formation (Weaver, 2015; Whisner et al., 2016). Calcium itself supports the growth of *Bifidobacterium* species that are associated with substantial alleviation of asthma (Chaplin et al., 2016).

Magnesium influences the diversity of gut microbiota (Bielik and Kolisek, 2021) where optimal levels of magnesium are favorable for gut microbiome homeostasis. Elevated magnesium in rats leads to overgrowth of proinflammatory phyla *Proteobacteria*, along with *Parabacteroides*, *Victivallis*, and *Butyricimonas*, whilst normal magnesium levels promote higher diversity of the gut microbiota (Garcia-Legorreta et al., 2020). Interestingly, deficiency or excessively high levels of copper have been associated with chronic inflammation. Deficiency of other elements such as selenium and zinc on the other hand, have been correlated with asthma. Other elements such as manganese show contradictory association with asthma pathogenesis (Zajac, 2021).

## 6 Drugs and vaccines

Alterations to the gut/lung microbiomes affects asthma symptoms and adds another layer of complexity to asthma pathogenesis. Unfortunately, asthma patients are often treated with medications that directly impair the gut and airway microbiota. Although some of these medications are recommended for asthma control, precise regulation and control are needed as misuse and overuse of them could be less beneficial or even harmful.

### 6.1 Antibiotics

Since their discovery, antibiotics have saved countless lives. However, misuse and overuse of these medications can result in the development of bacterial resistance, microbial dysbiosis and increased risk of diseases including diabetes, obesity, allergies and asthma (Blaser, 2016). Studies have shown that asthma development positively correlates with antibiotic usage during pregnancy and periods of life when antibiotic prescription is more frequent (Penders et al., 2006; Kozyrskyj et al., 2007; Marra et al., 2009; Martel et al., 2009; Murk et al., 2011; Penders et al., 2011; Risnes et al., 2011; Hoskin-Parr et al., 2013; Arrieta et al., 2015; Bookstaver et al., 2015; Blaser, 2016; Castro-Rodriguez et al., 2016; Loewen et al., 2018; Ni et al., 2019; Donovan et al., 2020; Zhong et al., 2021). In fact, international guidelines for asthma recommend against antibiotic usage in infants and young children (Global Initiative for Asthma, 2021). However, antibiotics are commonly prescribed to children (Hales et al., 2018), and given to children with asthma at a greater rate than children without asthma (Fong et al., 2021), sometimes without any justification (Paul et al., 2011).

Experimental studies have demonstrated that antibiotic use increases asthma symptom severity and reveal some of the immunologic mechanisms involved (Russell et al., 2012; Kim et al., 2014; Yang et al., 2019; Alhasan et al., 2020). Antibiotics reduce CD4^+^FoxP3^+^ T regulatory cells in the colon (Russell et al., 2012) and lung-draining lymph nodes (Adami et al., 2018), decrease cecal levels of SCFAs (Alhasan et al., 2020), promote M2 macrophage polarization in the lungs (Kim et al., 2014), leading to long-lasting alterations to the gut and lung microbiome richness, evenness, and composition (Jernberg et al., 2010; Yang et al., 2019). Additionally, expansion of fungi *Wallemia mellicola* and *Candida albicans* in the gut after antibiotic treatment exacerbates the airway inflammation after allergen exposure in mice (Noverr et al., 2004; Skalski et al., 2018). Similarly, antifungals induce intestinal dysbiosis and enhance allergic airway inflammation through gut-lung crosstalk (Wheeler et al., 2016; Li et al., 2018). Moreover, antibiotics affect immune responses to respiratory bacterial and/or viral infections associated with asthma exacerbations (Flores-Torres and Samarasinghe, 2022), including *S. pneumoniae* (Schuijt et al., 2016; LeMessurier et al., 2019) and influenza virus (Abt et al., 2012; LeMessurier et al., 2019).

The impact antibiotics have on the microbiome has been evaluated in humans. Azithromycin, for example is frequently used in low doses to reduce airway inflammation in chronic diseases like COPD and bronchiolitis obliterans. It is one of the most commonly prescribed antibiotics to pediatric patients and known to reduce the intestinal bacterial diversity and composition in children (Doan et al., 2017; Oldenburg et al., 2018). Smokers’ lung microbiota show a reduction in alpha-diversity after azithromycin treatment although the overall bacterial load may not change (Segal et al., 2017). Antibiotic-induced reductions in beneficial gut commensals like *Bifidobacterium* species and Actinobacteria may then permit the outgrowth of potentially pathogenic Firmicutes, Proteobacteria, and Bacteroidetes (Korpela et al., 2016; Reyman et al., 2022), some of which are linked to asthma exacerbations (Earl et al., 2015). Much remains unknown about the impact of antibiotics on the airway microbiome in the context of asthma, patient demographics, and lifestyle (Hufnagl et al., 2020).

### 6.2 Corticosteroids

Although current asthma guidelines recommend the use of corticosteroids to control asthma symptoms and exacerbations (Global Initiative for Asthma, 2021), there is evidence suggesting that their use is associated with increased risk of respiratory infections in asthma patients (McKeever et al., 2013; Qian et al., 2017) and alterations to the airway microbiome (Hartmann et al., 2021). For example, steroid-resistant patients present bacterial enrichment in *Microbacteriaceae* and *Pasteurellaceae*, families while steroid-responsive patients have enrichment of *Streptococcaceae*, *Fusobacteriaceae*, and *Sphingomonodaceae* in the upper RT microbiome (Durack et al., 2017). Steroid-resistant asthma patients also present an airway expansion of specific Gram-negative bacteria, which are suggested to trigger TAK1/MAPK signaling and induce corticosteroid resistance (Goleva et al., 2013). Patients receiving steroid therapy have increased fungal load in the BAL (Fraczek et al., 2018), with greater abundance of *Fusarium* and *Mortierella*, and reductions in *Wallemia*, *Alternaria* and *Aspergillus* compared to patients not on corticosteroids (Huang et al., 2020).

Studies investigating airway dysbiosis in asthma patients considered corticosteroids as confounders to disease-induced changes on the microbiome. Indeed, patients with asthma do present with airway dysbiosis independent of corticosteroid treatment (Durack et al., 2017). However, there is evidence suggesting that corticosteroids can drive further alterations in the asthma patient microbiome, as corticosteroids associate with decreased abundance of Bacteroidetes, Fusobacteria and *Prevotella* species, and with increased abundance of Proteobacteria (Denner et al., 2016; Durack et al., 2017). Neutrophils are resistant to corticosteroid-induced apoptosis (Saffar et al., 2011) although patients with neutrophilic and paucigranulocytic asthma may be treated with higher doses of corticosteroids (Cowan et al., 2010). Therefore, effects of corticosteroids may amalgamate with underlying changes to the microbiome in patients with neutrophil dominant asthma phenotypes to cause an overall reduction in bacterial diversity and richness (Taylor et al., 2018).

### 6.3 Vaccination

Multiple studies have shown that microbiome perturbation alters the vaccine-induced antibody responses (Oh et al., 2014). Due to the emphasis on the vaccination on the gut microbiota (de Jong et al., 2020), less is known about the vaccine efficacy during dysbiosis in other niches including the airways. Although the live-attenuated influenza vaccine (LAIV) is the only intranasally administered vaccine, other intranasal vaccines such as severe acute respiratory syndrome coronavirus 2 (SARS-CoV-2) are under development (de Jong et al., 2020). The LAIV immunogenicity is now reported to be dampened by Streptococcal colonization in humans (Carniel et al., 2021), which is interesting since *S. pneumoniae* colonization rate is high in children (Bogaert et al., 2004), and asthma patients have a higher carriage compared to healthy individuals (Jounio et al., 2010). These data suggest that LAIV could be less effective among asthma patients. Additionally, LAIV is contraindicated in wheezing children with precautions in 5 year old asthma patients (Grohskopf et al., 2020) although the LAIV has been proven safe and effective by others (Flores-Torres and Samarasinghe, 2022).

## 7 Conclusion

The risk factors for the pathogenesis and severity of asthma are multifactorial and include among other factors, environmental pollutants, obesity and poor nutrition, overall poor socioeconomic status, and even repeated exposure to toxic stress. Whether race contributes to asthma severity and outcomes more as a biological factor or a social construct is unclear, but what is clear is that higher prevalence, higher severity, and worse outcomes are associated with the Black race as defined in the literature. Better classification of these relationships will provide a blueprint for more effective clinical interventions. Drastic measures to reduce complications of childhood obesity, including bariatric surgery, have decreased the frequency and severity of asthma exacerbations. Although not discussed in detail here, environmental pollutants including particulate matter are also variable depending on housing location of patients. For example, areas of highest asthma incidence are often located in zip codes adjacent to large cities, airports, or factories. All these components can impact the host microbiome which may imprint the next generation as well (Graphical Abstract).

A multitude of factors contribute to poorly controlled asthma that we see in our city of Memphis. While it is true that the lung microbiome is adversely affected by childhood infections, Tennessee is the sixth highest state in the nation for outpatient antibiotic prescribing practices with 1,169 prescriptions per 1,000 patients. Although the exact mechanism of how antibiotics negatively affect the lung microbiome and why they are overprescribed remain unknown, there is supporting data that a healthy gut microbiome results in better vaccine effectiveness against viral infections. It is reasonable to conclude that the overuse of antibiotics is actively contributing adversely to asthma severity we see in our community, with our emergency department caring for approximately 8,000 asthma related patient care visits per year.

The future of scientific study into new therapeutics and possibly, one day, gene directed therapy is exciting. Development of targeted treatments for various asthma subtypes seems promising, as does the development of immune and monoclonal antibody therapeutics, but this seems a lofty goal when many of the patients suffering with the most treatment-resistant asthma do not even have access to their own beta agonist inhaler. Thankfully, inhaled corticosteroids are relatively inexpensive by comparison and have been the mainstay of chronic asthma management. The exact effect of inhaled corticosteroids on the lung microbiome has yet to be fully understood, however research up to this point suggests that such therapy may be beneficial for some patients but harmful for others as it negatively affects the lung microbiome.

## Figures and Tables

**FIGURE 1:**
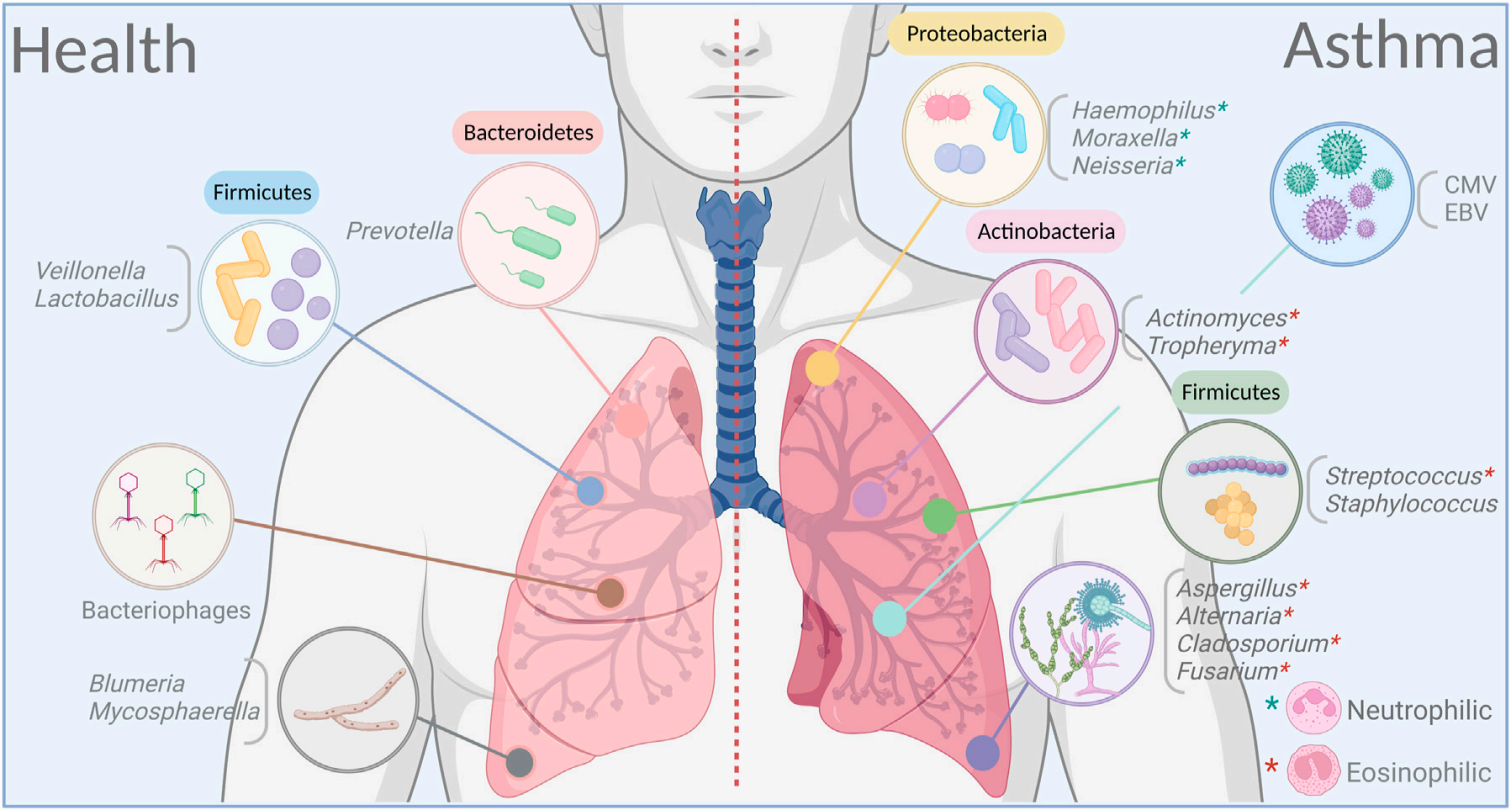
Dominant bacteria, viruses and fungi in the airways in health and asthma. Certain microbial families dominate in lungs on healthy individuals to maintain a balance between varying ecosystems and remain in a symbiotic relationship with the host. This balance is rapidly broken during altered immune states in response to an exogenous trigger as in asthma. Dysbiosis, whether by cause or as effect, is a prominent feature in asthma, and may have a long-lasting impact on both the endogenous microbiome and the host. Figure prepared on BioRender.
